# The Urgent Need for Robust Coral Disease Diagnostics

**DOI:** 10.1371/journal.ppat.1002183

**Published:** 2011-10-20

**Authors:** F. Joseph Pollock, Pamela J. Morris, Bette L. Willis, David G. Bourne

**Affiliations:** 1 Australian Institute of Marine Science, Townsville, Australia; 2 Grice Marine Laboratory, College of Charleston, Charleston, South Carolina, United States of America; 3 Hollings Marine Laboratory, Charleston, South Carolina, United States of America; 4 ARC Centre of Excellence for Coral Reef Studies, School of Marine and Tropical Biology, James Cook University, Townsville, Australia; 5 Belle W. Baruch Institute for Marine and Coastal Sciences, University of South Carolina, Georgetown, South Carolina, United States of America; University of California San Diego, United States of America

## Abstract

Coral disease has emerged over recent decades as a significant threat to coral reef ecosystems, with declines in coral cover and diversity of Caribbean reefs providing an example of the potential impacts of disease at regional scales. If similar trends are to be mitigated or avoided on reefs worldwide, a deeper understanding of the factors underlying the origin and spread of coral diseases and the steps that can be taken to prevent, control, or reduce their impacts is required. In recent years, an increased focus on coral microbiology and the application of classic culture techniques and emerging molecular technologies has revealed several coral pathogens that could serve as targets for novel coral disease diagnostic tools. The ability to detect and quantify microbial agents identified as indicators of coral disease will aid in the elucidation of disease causation and facilitate coral disease detection and diagnosis, pathogen monitoring in individuals and ecosystems, and identification of pathogen sources, vectors, and reservoirs. This information will advance the field of coral disease research and contribute knowledge necessary for effective coral reef management. This paper establishes the need for sensitive and specific molecular-based coral pathogen detection, outlines the emerging technologies that could serve as the basis of a new generation of coral disease diagnostic assays, and addresses the unique challenges inherent to the application of these techniques to environmentally derived coral samples.

## The Need for Improved Coral Disease Diagnostic Tools

The world's coral reefs are in decline, with hard coral cover on Caribbean reefs decreasing by an average of 80% in the last 30 years [1] and Indo-Pacific reefs suffering an estimated coral cover loss of 50% over the same period [2]. The causes of these declines are diverse and complex, including water pollution, habitat destruction, overfishing, invasive species, and global climate change [3]–[5]. In recent years, coral diseases have also emerged as a significant threat to the world's coral reef ecosystems [6], [7]. Since the first coral disease was described in 1973, evidence from field studies documenting the population and community-level impacts of disease on coral reef ecosystems worldwide has been accumulating (reviewed in [8]) [9]–[14] and it is now clear that coral diseases have the potential to cause widespread mortality and significantly alter reef community structure (e.g., [9], [15]–[17]).

**Figure 1:**
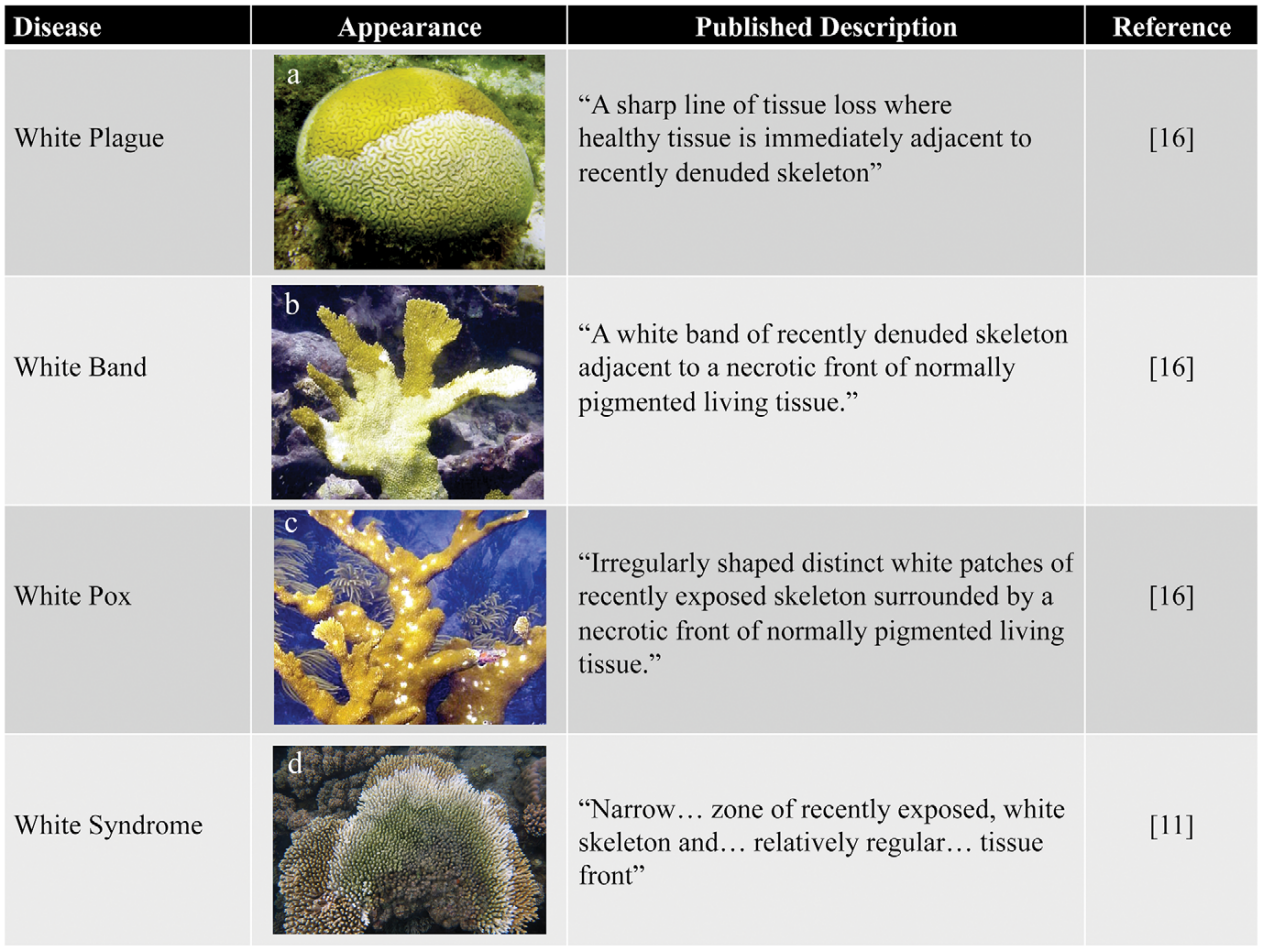
Examples of white diseases affecting Scleractinian corals. (a) White plague in *Diploria labyrinthiformis*; (b) white band in *Acropora palmate*; (c) white pox in *A. palmate*; and (d) white syndrome in *A. millepora*. Photos courtesy of Ernesto Weil.

Despite the serious threat that coral diseases pose to the health of reef ecosystems globally, little is known about many of these diseases, including their etiologies, transmission dynamics, and the steps that can be taken to prevent, control, or reduce their impacts. This work has been frustrated by the inability to determine etiological agents for many diseases (see Box 1), insufficient diagnostic tools, and limited application of established biomedical diagnostic methods [18]. Current diagnostics focus on documenting disease signs *in situ*, describing macroscopic characteristics such as species affected, extent and pattern of tissue loss [19], presence and appearance of microbial mats [15], abnormal coloration [20], or skeletal anomalies [21]. Corals display few macroscopic signs indicative of stress and consequently an array of maladies, including environmental stress, predation, and infectious disease, are often manifested as a paling or sloughing of the coral tissue. For example, more than six “white” diseases, which are characterized by a spreading zone of tissue loss, exposing white coral skeleton directly adjacent to asymptomatic coral tissue, have been described in the Caribbean alone (Figure 1) [16]. Because of their nearly identical appearance, several of these diseases (e.g., white plague I and white plague II) are differentiated almost exclusively by the rate of lesion progression over the infected colony [16]. Such difficulties have resulted in cases of misidentified diseases, repeated name changes for the same disease [22], and even classification of predation scars as disease [10], [23]. Currently, it is uncertain how many distinct coral diseases exist worldwide; in two articles published in the same year, one report identified 18 diseases [16], whereas another put the number at 29 [8]. This confusion underlines the need for more robust coral disease diagnostic methods.

**Box 1. The Need for Surrogate Models to Study Coral Disease**

The development of effective coral disease diagnostics requires efficient methods for identifying microbial drivers of disease and an understanding of the pathogenesis of identified disease agents. In the field of human health, investigations using surrogate hosts, including the common rat (*Rattus norvegicus*), the house mouse (*Mus musculus*), and the common fruit fly (*Drosophila melanogaster*), have been integral to unraveling the intricate interactions between host, pathogen, and environment that lead to disease. A few coral species, including *A. millepora* in the Indo-Pacific and *A. palmata* in the Caribbean, have emerged as “lab rats” for the study of coral genetics [88], [89], physiology [90], [91], and health [92], [93]. However, our ability to study coral disease pathogenesis in the laboratory has been limited by: the complexity of the coral holobiont, which comprises animal, dinoflagellate, and microbial partners; a poorly understood coral immune system (see Box 2); and difficulties associated with sourcing and rearing these sensitive and often protected species [94]. For example, since *A. palamata*, which was once the dominant coral species throughout much of the Caribbean, was added to the IUCN Red List of critically endangered species [95], acquiring specimens for experimentation has become much more difficult both logistically and morally. By focusing disease investigations on surrogate models for the coral host, researchers may be able to overcome these limitations while still gaining valuable insights into the complex interactions between host, environment, and pathogen that lead to disease in corals.

In search of alternative surrogates for the coral animal, researchers have explored cnidarians in the class Anthozoa, including the *Symbiodinium*-harboring, tropical anemone *Aiptasia sp.* [96], as well as more distantly related hydrozoans, such as freshwater *Hydra* species [97]. Research on *Aiptasia*, for example, has provided insights into the physiological responses of anthozoans and their algal symbionts to thermal stress and bleaching [98], [99], and *Hydra* species have been used to explore the development and maintenance of cnidarian-associated bacterial communities [97]. These readily available, easily reared, and phylogenetically closely related coral analogues could also provide insights into the role of coral-associated microbes as mutualistic, commensal, or pathogenic and potentially reveal the functional pathogenesis mechanisms of identified pathogens.

Once potential pathogens are identified and their virulence mechanisms determined in surrogate hosts, researchers must confirm these findings within the complex coral holobiont. Captive-bred coral juveniles and laboratory-maintained *Symbiodinium* cultures provide easily replicated and environmentally responsible alternatives to wild-harvested adult colonies for laboratory-based experimentation. Both brooding and mass spawning corals provide thousands of genotypically similar coral juveniles from just a single pair. Although many spawning species breed during short periods each year [100], limiting the availability of juveniles to researchers, some brooding corals release gametes much more frequently [101]. Furthermore, juveniles of some coral species can be maintained *Symbiodinium* free for weeks, allowing researchers to control the algal symbionts they uptake [102]. Many research laboratories currently possess pure and mixed cultures of coral-derived *Symbiodinium*, which could be used to seed juveniles or directly test the effect of putative pathogens on the *Symbiodinium* themselves. An experimental system comprised of *Symbiodinium* cultures, asymbiotic, and symbiotic juveniles (or other *Symbiodinium*-harboring cnidarians such as *Aiptasia*) would allow researchers to tease out the targets of specific pathogens within the coral holobiont. By focusing research on a few, well-chosen model systems, researchers will be better able to identify potential pathogens and study their virulence mechanisms in an efficient, environmentally friendly, and easily comparable manner.

In recent years, an increased interest in coral microbiology, in combination with the application of histology and biomedical approaches, has revealed several bacterial species linked to coral disease lesions [6], [24], [25]. Debate exists as to the primacy of a compromised coral host versus opportunistically proliferating bacteria in causing coral diseases [26], [27]. However, since disease is classically considered to be the outcome of interactions among a causative agent, susceptible host, and the environment (e.g., [28]), debating the status of an etiological agent as either a primary or secondary pathogen is diversionary and does not negate the need to understand its role in pathogenesis [18], [29]. While coral immunity plays a critical role in maintaining coral health and indicators of coral immune status can provide insight into the health state of the coral host (summarized in Box 2), this is not the focus of this review. Here we discuss the use of identified coral pathogens or disease indicators as targets for a new generation of sensitive and highly specific, molecular-based diagnostic assays that can begin to address many of the basic questions that plague the field of coral disease research.

**Box 2. The Diagnostic Potential of Coral Immunity**

Recent reviews have highlighted immunological indicators of coral stress and disease [16], [103], [104], thus here we briefly discuss coral immune response as a proxy for disease susceptibility and as an indicator of past or present exposure to pathogens or other stressors (e.g., high water temperatures, excessive UV exposure). Corals, like other invertebrates, are limited to innate immunity [105], which is defined as the ability of certain cells and cellular mechanisms to defend the host from infection by other organisms in a nonspecific manner [106]. Much work is currently focused on the use of coral host factors and immunological responses as indicators of coral stress and disease [107], [108]. For corals and other marine invertebrates, phagocytosis provides the first line of cellular defense [109], [110]. In response to invasion by a pathogen, corals increase production of motile phagocytic cells, also known as amoebocytes, that migrate from healthy coral tissues to the site of infection and either attack the invading pathogens via phagocytosis or contribute to healing and regeneration of the damaged tissue [104], [107]. Histology is a well-established technique for detection and quantification of amoebocytes in the coral host. The examination of specially stained histological slides has been used to detect amoebocyte accumulation in response to a range of insults including sedimentation [111], skeletal anomalies [112], and disease [107].

Exposure of corals to pathogens also induces production of antibiotic compounds, which may instill some resistance against invading microbes. Gorgonian corals have been shown to resist infection by the fungus *A. sydowii*, the causative agent of aspergillosis, through the production of antifungal agents that inhibit germination of *A. sydowii* spores [113], [114]. White syndrome and yellow band disease have also been shown to induce antimicrobial activity in scleractinian corals [115], [116]. Methods exist for the detection of antimicrobial residues in animals [117] and analogous assays could easily be adapted for corals.

Recent investigations have revealed the melanization cascade to be an integral component of coral immunity. The melanization cascade involves the production of prophenoloxidase (PPO), which is involved in wound healing, encapsulation, and disease resistance [107], [103]. PPO serves as the precursor molecule of phenoloxidase (PO), which is activated by proteases during active pathogen invasion and in turn induces the deposition of melanin, the endpoint of the cascade and a potent physiochemical barrier [107], [108]. Melanin has antimicrobial and cytotoxic attributes, and therefore its presence in stressed and diseased corals implies the activation of innate immune responses. Assays to detect PO and melanin in coral samples have been developed [108], [112], which could be included in future disease studies [103]. The ability to detect and quantify amoebocytes, antimicrobial compounds, melanin deposits, and the precursors of melanization, including PPO and PO, will provide proxies for immune response in corals. Although immune response is not a direct indicator of disease, these parameters could be used to assess coral health, disease susceptibility, and past or present exposure to pathogens or other stressors.

## Benefits of Pathogen-Specific Detection Tools

In this section, we highlight the role that specific and sensitive pathogen detection will play in advancing our understanding of the etiology, spread, and ultimately management of coral diseases.

### Detecting Shifts in Coral-Associated Microbial Communities

The coral holobiont comprises a complex association between the coral animal and its microbial partners, including symbiotic dinoflagellates (zooxanthellae) [30], bacteria [14], [31], archaea [32], [33]), viruses [34], endolithic algae [35], and fungi [36]. Numerous studies have examined these associations in both healthy and stressed corals and it has been suggested that shifts in microbial communities can act as indicators of coral stress [37]–[39]. For example, Pantos et al. [37] demonstrated bacterial community shifts throughout the entire coral colony, even when just a small part of the colony showed signs of disease, and Bourne et al. [38] reported shifts in coral-associated microbial communities well before the appearance of visual signs of thermal bleaching. Using metagenomic approaches, Vega-Thurber et al. [39] demonstrated functional gene shifts, including an increased abundance of virulence genes, in coral microbial partners during temperature, nutrient, and pH stress, although it should be noted that this study did not quantify expression of these genes. Additionally, Kimes et al. [40] observed significant differences in biogeochemical cycling-related genes between healthy and yellow-band infected *Montastraea faveolata* colonies. These community-level bacterial profiling approaches facilitate diagnosis at the earliest stages of infection when mitigation measures would be most effective [41]. Therefore, the development of rapid and sensitive assays to monitor coral-associated microbial communities as proxies for coral health should be a research focus.

### Better Understanding of Disease Etiology

While some coral diseases are tightly linked with the presence of a specific pathogen, the causes of many other diseases and disease-like syndromes remain elusive [41]. Better tools with high specificity for target pathogens would enable investigations of the circumstances under which microbes that are normally found on corals become pathogenic and the conditions and mechanisms that trigger a switch from commensal or neutral to pathogenic. Moreover, there are cases where bacterial species, which were linked to specific diseases in early studies, no longer elicit the same response or are not associated with disease signs, potentially indicating development of disease resistance [6], [42]. For example, *Vibrio shiloi*, which was initially identified as the agent responsible for annual bleaching of the Mediterranean coral *Oculina patagonica*, no longer appears to cause bleaching in this coral species [27], [43]. Additionally, *Aspergillus sydowii*, which was shown to cause disease in gorgonians, has also been found on healthy coral colonies, leading Toledo-Hernandez et al. [44] to raise questions about its role in disease onset. The development of tools to detect and quantify putative pathogens in both controlled laboratory experiments and environmentally derived samples will help to establish the etiology of specific coral diseases and clarify the role of individual microbes in the onset of disease lesions. Once the link between a specific microbial entity and lesion onset is established, pathogen-specific assays can provide information on all aspects of the disease onset process.

### Monitoring Pathogen Load

Emerging evidence suggests that the abundance of coral pathogens varies on reefs throughout the year and within coral hosts during the course of infection [45]–[47]. The ability to quantify pathogen load in coral and environmental samples will allow researchers and reef managers to gauge the health status of individual corals, assess the impact of environmental parameters (e.g., temperature, nutrient load, sedimentation rate) on pathogen load, and better predict large-scale disease outbreaks. Some efforts have been made to establish links between environmental parameters and coral disease prevalence. Using high-resolution satellite datasets and long-term coral disease surveys, Bruno et al. [2] established a link between coral disease outbreaks and warm temperature anomalies at sites with high coral cover. By monitoring bacterial communities in situ, Vezzulli et al. [47] also discovered a link between mass mortality events of the coral *Paramuricea clavata* and seawater temperatures, chlorophyll concentrations, and the presence of culturable *Vibrio* spp. in the surrounding seawater. Tools for monitoring pathogen density would provide a deeper understanding of how pathogen load and virulence respond to natural (e.g., seasonal, El Niño/La Niña) and anthropogenic (e.g., pesticide and nutrient influx, sedimentation) fluctuations, allowing researchers and managers to closely follow these dynamics and model pathogen response to environmental change.

### Identifying Pathogen Sources, Vectors, and Reservoirs

It is currently unclear if the emergence of coral diseases on reefs is associated with the introduction of pathogenic organisms, or whether potentially pathogenic microbes are a normal component of reef ecosystems that increase in virulence because of altered environmental conditions and/or reduced host resistance. To better understand the dynamics of coral disease outbreaks and ensure that they are effectively managed, information regarding pathogen sources, vectors, and reservoirs is needed. Pathogen sources are the avenues through which a pathogen enters the environment, reservoirs are biotic or abiotic entities that harbor a pathogen, and vectors are living entities that do not cause or suffer from a disease, but transmit a pathogen from one host to another [28]. The identification of the marine fireworm as the winter reservoir and spring/summer vector of the coral pathogen *V. shiloi* nicely demonstrates the utility and importance of molecular-based pathogen detection techniques in the study of coral epidemiology [46].

### Better Informed Management Decisions

To effectively manage coral disease outbreaks, a deeper understanding of the causes of observed diseases, how they are spread between colonies and populations, and how environmental parameters influence pathogen virulence and host susceptibility to infection is required [48]. Tools that increase our capacity to establish links between disease signs and the presence of specific microbial agents will improve coral disease classification and diagnosis. These capabilities will help reef managers to discern the threats that impact the occurrence, prevalence, and severity of diseases so their sources can be identified and possibly reduced through better management practices [48]. For example, habitat degradation, poor water quality, and warming seas are often speculated as causes of the recent rise in coral diseases [13], but few studies have directly linked specific factors with increases in coral disease. By understanding the relationship between various stressors and the occurrence of coral diseases, managers may be able to identify potential threats in a timely manner and develop strategies to lessen their impacts [48]. Several biological controls for coral diseases, including bacteriophage therapy and probiotic addition, have recently been proposed [41], [49]. Pathogen-specific diagnostics could be used to identify where and when these controls should be implemented and also assess their efficacy. In order to assist resource managers to combat disease epizootics, prevent future outbreaks, and reduce the time needed for recovery, the development of sensitive, specific, and robust coral disease diagnostics should be an essential research priority [7].

## Pathogen Detection Methods

Effective diagnostic tools must be sensitive, reproducible, and specific in their detection of targeted microbial organisms. In the field of human pathogen detection, culture and colony counting, immunology, and nucleic acid-based methods are most commonly used [50]. Here, we provide a brief overview of these techniques and evaluate their potential for coral pathogen detection (summarized in Table 1].

**Table 1:** Summary of pathogen detection techniques and molecular diagnostics.

Technique	Principle	Advantages	Disadvantages	Used for Oral Pathogen Detection?
Culture and colony counting	Samples are plated onto selective growth media, incubated, and resulting colonies counted	▪ Well established in human disease diagnosis▪ Low cost	▪ Extensive development and testing of selective media▪ Long wait time for test result▪ Low sensitivity	[47], [55]
Antibodies	Samples are hybridized with pathogen-specific antibodies and antibody/antigens complexes are detected	▪ Well established in human disease diagnosis▪ High specificity of monoclonal antibodies	▪ Monoclonal antibodies are slow to develop▪ Low specificity of polyclonal antibodies▪ Antibody-producing cell lines difficult to maintain	[56]
FISH	Samples are hybridized with custom-synthesized nucleic acid probes attached to fluorescent reporter molecules and then visualized under fluorescence microscopy	▪ Use of different fluorescent reporters allows for simultaneous detection of multiple microbes▪ Allows localization and visualization of microbes within host tissue	▪ Low specificity of FISH probes▪ Time consuming and labor intensive processing▪ Autofluorescence of zooxanthellae and coral necessitates specialized imaging microscopy equipment	[27], [46], [61], [62]
PCR	Samples are subjected to PCR amplification with specific primer sets, then PCR products are separated by gel electrophoresis and visualized	▪ High sensitivity▪ High specificity	▪ Not quantitative▪ High contamination risk▪ Potential for nonspecific primer binding and amplification	[51], [63]
Real-time qPCR	Samples are subjected to PCR amplification incorporating a fluorescent reporter that emits a signal proportional to the quantity of PCR product synthesized	▪ High sensitivity▪ High specificity▪ Low contamination risk▪ Quantitative results	▪ High cost▪ Requires specialized thermocycler	[77]

### Culture-Based Detection

The culture and plating method is the oldest bacterial detection technique and remains a cornerstone of human pathogen detection. This method involves plating of samples onto selective growth media followed by an incubation period and then colony counting. Specialized growth media can contain inhibitors of nontarget species/strains, substrates that only the targeted microbe can degrade, and/or substances that confer a particular color to the growing colonies [50]. However, selective media take time to develop and test, and even when selective media are available for a pathogen of interest, culture and plating techniques are excessively time consuming and less sensitive than immunologic or genetic-based techniques [51], [52]. For corals, standardized diagnostics based on culture-dependent methods are limited, largely due to the lack of selective media capable of promoting growth of specific pathogens amongst the highly complex, diverse, and abundant microbial populations associated with compromised coral tissues. For example, selective media, such as thiosulfate citrate bile sucrose (TCBS), have been developed to discriminate *Vibrio* bacteria from other bacterial species. While known coral pathogens, including *V. coralliilyticus* and *V. shiloi*, can be grown on TCBS agar, they cannot be effectively discriminated from other *Vibrio* species [53] that coexist within the coral holobiont. In order to overcome this limitation, Ritchie et al. [54] developed a method to discriminate potential bacterial invaders from normal residents of the coral holobiont by including sterile coral mucus into the growth media. This innovative approach is based upon the assumption that symbiotic coral-associated bacteria will be resistant to the antibiotic properties of coral mucus while opportunistic pathogens will not. By comparing the bacterial strains growing on mucus-treated media plates to those growing on control media plates, it is theoretically possible to separate coral associated bacterial residents from potentially invasive visitors. While this technique is useful for identifying potential pathogens, the processes is extremely time consuming, requiring isolation of individual colonies, PCR amplification, and sequencing, and does not allow specific detection at the single pathogen level.

In a few cases, culture protocols have been developed to selectively grow specific coral-associated microbes. For example, Sutherland et al. [55] developed a technique to isolate *Serratia marcescens*, the presumed etiological agent of white pox in the Caribbean, involving two subsequent colorimetric culture steps followed by inoculation onto nonselective media. Interestingly, this method revealed human sewage to be a likely source of the pathogen on reefs in the Florida Keys. Where appropriate selective media exist, most probable number (MPN) methods can be used to estimate the concentration of bacteria [47]. MPN involves serially diluting samples into appropriate media, further dividing these dilutions into replicate aliquots, culturing, and assigning a binomial (growth versus no growth) score to the resulting cultures. This method can be used to estimate the concentration of certain bacterial groups in a given sample; however, the dilution and culturing steps can be time consuming and reproducibility is often an issue. Due to the high diversity of microbes present in coral samples, lack of appropriate media for many coral pathogens, and the low sensitivity and long processing time required, culture-based diagnostic methods are not the ideal platform for coral pathogen detection.

### Immunology-Based Detection

The use of antibody technology is well established in human medical diagnostics and has been applied with some success to the detection of coral pathogens. Immunology-based pathogen monitoring involves the production of either polyclonal or monoclonal antibodies and the detection of antibody/antigen complexes that indicate the presence of the targeted pathogen within a sample. Specific anti-*V. shiloi* antibodies have provided insight into the dynamics of pathogen invasion and spread within the *O. patagonica* coral host, suggesting a temperature-dependent host defense against the pathogen [56]. However, immunology-based techniques can only be developed once specific pathogens have been identified and successfully cultured (see Box 1). Furthermore, polyclonal antibodies often have low specificity [57] and highly specific monoclonal antibodies are generally slow to develop and expensive to produce and maintain. While immunology-based coral pathogen detection is feasible once specific pathogens have been successfully isolated, the cost and effort required to develop and maintain antibody-producing cell lines may limit its utility in routine monitoring. However, if adapted into routine assays such as ELISA, common in many human health targeted kits such as pregnancy tests [58], [59], this approach has the potential to provide rapid coral pathogen detection.

### Nucleic Acid-Based Detection

Nucleic acid-based techniques using molecular probes and/or PCR offer an appealing alternative to culture and immunology-based methods because of their potential for high specificity and sensitivity. Here we discuss the utility of fluorescent in situ hydridization (FISH) and PCR-based techniques in coral pathogen detection.

### 

#### Fluorescent in situ hybridization

FISH allows identification, localization, and visualization of individual microbial cells within healthy and diseased tissue [60] by targeting these microbes with custom-synthesized nucleic acid probes attached to fluorescent reporter molecules. Ainsworth et al. [27], [61], [62] utilized FISH to assess the microbial composition of diseased corals in the Mediterranean [27], Red Sea [62], and on the Great Barrier Reef [61]. *V. shiloi*-specific FISH probes also revealed the marine fireworm *Hermodice carunculata* as the reservoir and transmission vector of this coral bleaching pathogen [46]. While these studies provide useful information on the spatial arrangement of microbes in healthy and diseased tissue, the low specificity of FISH probes can limit their utility in accurately detecting pathogenic microbes beyond the genus level. In addition, the method is time consuming, labor intensive, and requires specialized imaging microscopy equipment. Extensive processing of samples may also result in the loss of loosely attached microbes including the pathogen cells themselves. Therefore, although helping to elucidate disease etiology, the utility of FISH as a routine coral disease diagnostic is limited.

#### PCR-based methods

PCR-based methods allow high sensitivity and specificity by targeting and amplifying short nucleic acid (DNA or RNA) sequences within the genomes of coral-associated microbes [63]. These methods are far less time consuming than most culture or immunology-based approaches, yielding results in hours rather than days or even weeks with some culture-based techniques [64]. Several community-level PCR techniques, including denaturing gradient gel electrophoresis (DGGE) [38], terminal restriction fragment length polymorphism (T-RFLP) [65], [66], automated ribosomal intergenic spacer analysis (ARISA) [67], 16S rRNA clone libraries [38], and microarrays [40], [68] have provided insights into the microbial communities associated with healthy and stressed corals. Although this information can be used to detect shifts in community structure, these changes cannot be linked to specific pathogens. Even when specific pathogens have been identified, standard PCR-based methods do not provide accurate quantification of individual microbial species/strains.

#### Real-time quantitative PCR

The combination of high sensitivity and specificity, low contamination risk, ease of performance, and speed make real-time, quantitative PCR (qPCR) technology an appealing option for specific coral pathogen detection [69]. qPCR allows for accurate quantification of microbe densities by incorporating a fluorescent reporter in the PCR reaction that emits a signal proportional to the quantity of PCR product. This information can then be used to infer the amount of target gene and relative number of pathogen cells in a given sample [70]–[72]. qPCR assays have been designed for a number of bacterial [73], [74], fungal [75], and viral [76] pathogens. For example, a real-time PCR assay was developed to detect *V. penaeicida* in the prawn *Litopenaeus stylirostris* and aquaculture facilities in New Caledonia [64]. This single-day assay provided a research tool for understanding the dynamics of this pathogen within aquaculture facilities and served as a decision-making tool for prawn farmers. Analogous assays to detect and quantify coral pathogens in environmentally derived samples are beginning to emerge. For example, Pollock et al. [77] developed a qPCR assay to detect the identified coral pathogen *V. coralliilyticus*. This technique, which is capable of detecting the bacterium at concentrations as low as 1 CFU ml^−1^ in seawater and 10^3^ CFU cm^−2^ on coral fragments, is currently being used to investigate the epidemiology of *V. coralliilyticus*, including information on its distribution and role in the initiation and spread of white syndrome lesions in the Indo-Pacific. This assay represents the first application of qPCR technology for the detection of an established coral pathogen.

**Figure 2:**
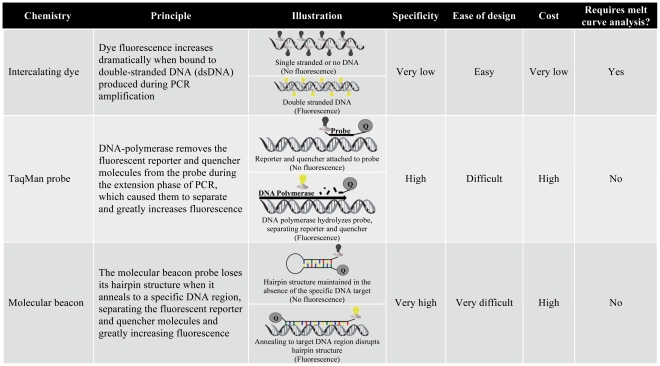
Summary of qPCR chemistries.

qPCR technologies fall into two broad categories on the basis of their fluorescence chemistries: (1) intercalating dyes and (2) oligonucleotide-specific probes (Figure 2). Intercalating dye technologies, such as SYBR Green, fluoresce as they anneal to the double-stranded DNA (dsDNA) that is synthesized during PCR amplification. As the quantity of dsDNA increases during subsequent PCR cycles, the fluorescence signal increases proportionally (illustrated in Figure 2) [78]. Oligonucleotide probe technologies, including TaqMan and Molecular Beacon, add an additional layer of specificity to the qPCR assay by incorporating a sequence-specific probe that must anneal to a particular region within the PCR amplicon for fluorescence (illustrated in Figure 2). Intercalating dyes are more commonly used than probe technologies because they are less expensive and work with traditional PCR primer sets, negating the time and labor-intensive design of specific probes. However, since intercalating dyes fluoresce in the presence of any dsDNA, they are not specific and must be accompanied by melting curve analysis to differentiate PCR products on the basis of length and G-C content [69]. Oligonucleotide-specific probes are more specific, but also more expensive and require the design of custom synthesized probes. Probe technologies also allow inclusion of several distinct primer/probe sets labeled with different colored fluorescent reporters in a single reaction, facilitating the simultaneous detection of several pathogens.

### DNA Target Selection

For the development of molecular diagnostic assays, the choice of a nucleic acid target is just as important as the platform used to detect it. To allow for high specificity, nucleic acid targets must be both well-conserved within the genome of the target species/strain and distinct from nontarget sequences. Therefore, a great deal of care must be taken in genetic target selection, primer/probe design, and assay optimization. Ribosomal and mitochondrial DNA are the most common targets for nucleic acid-based microbe detection because of their genetic stability and high copy numbers within cells [79]. However, other genes, including housekeeping genes and virulence factors that are present as only a single or limited copy number in the genome, may also serve as useful targets.

### 

#### Ribosomal genes

Several ribosomal RNA genes, including 16S, 18S, 23S, and internal transcribed spacer region (ITS) genes, have served as targets for nucleic acid-based detection. With the public availability of over 2 million 16S rRNA sequences (GenBank), spanning both the variable and more highly conserved regions of this ubiquitous bacterial gene, the 16S rRNA gene provides an obvious nucleic acid target. Since its introduction in the late 1980s, most FISH applications have targeted rRNA genes because of the large number of publically available sequences and its high copy number in bacterial cells [60]. However, low genetic divergence in closely related species/strains often hinders the utility of the 16S rRNA gene in differentiating beyond the genus level [80]. For example, the known coral pathogen *V. coralliilyticus* shares greater than 98% 16S sequence similarity with its closest phylogenetic neighbor, *V. neptunius* [80]. Although some variation (2%) exists between these closely related species, it is likely inadequate to design sufficiently specific primers and/or probes.

#### Genomic phylogenetic marker genes

The accelerated use of genetic sequencing as a means of differentiating closely related bacterial species and strains has led to the proliferation of sequence information from a large number of nonribosomal phylogenetic marker genes in a diverse sampling of microbial species. In some of the better-studied groups, such as the vibrios that contain four of the seven described coral pathogens, sequence data from several phylogenetic marker genes are available for all described species [80] and even multiple strains of the identified coral pathogen *V. coralliilyticus* [81]. This information is useful for selecting genes with the greatest discriminatory power based on phylogenetic reconstructions and also provides the raw sequence data to identify specific oligonucleotide sequences within these genes, which can be targeted by custom-designed molecular primers and probes.

#### Virulence factors

In the field of human medicine, an increasing number of molecular-based pathogen detection assays have targeted genes directly involved in virulence. For example, the thermostable direct hemolysin gene (*tdh*) has been used as a target for detection of the human pathogen *V. parahaemolyticus* and the gene is also inferred as a direct marker of its pathogenicity [82]. Similarly, the hemolysin gene (*vvh*) has been targeted for the specific detection of *V. vulnificus* in oysters [73]. Directly targeting strain-specific virulence factors provides a means of differentiating pathogenic and benign strains; by targeting a 2-kb fragment of the cytotoxin-coding gene (*rtxA*) unique to virulent strains of *V. cholerae*, Gubala [74] designed a qPCR-based assay capable of exclusively detecting potentially toxigenic strains.

Specific virulence factors have been described in two coral pathogens, the Zn-metalloprotease gene (*vcpA*) in *V. coralliilyticus* and the Toxin P gene in *V. shiloi*, both of which could serve as molecular targets [81], [83], [84]. As researchers develop a deeper understanding of the genetic basis of coral pathogen virulence, it is likely that more virulence targets will become available.

### Emerging Diagnostic Techniques

Application of the technological advances outlined above will undoubtedly enhance our ability to study coral diseases; however, a variety of new and emerging technologies will further revolutionize the field in decades to come. High resolution microarrays offer one method for rapid assessment of shifts in coral-associated bacterial community structure. For example, Sunagawa et al. [68] utilized a 16S rRNA gene microarray (PhyloChip G2) to characterize the bacterial community structures of asymptomatic and diseased corals and investigate the etiology of the observed disease. If known bacterial groups or indicator organisms are indentified that are important to coral health, these shifts can be used to infer potential changes in coral health or, additionally, detect identified pathogens associated with disease. Vega Thurber et al. [39] assessed changes in overall bacterial community structure and abundance of functional genes in response to environmental stressors using a 454 pyrosequencing platform. Comparative genomic approaches such as these will continue to provide insights into the bacterial community-level changes that accompany coral stress and potentially facilitate coral disease outbreaks.

Transcriptomic approaches also have great potential for the identification of organisms actively involved in the infection process as well as virulence genes controlling disease progression. To date, the application of RNA-based expression studies on diseased coral samples is limited, except for certain band diseases, such as black band disease [85], where the microbial mat can first be separated from the coral. This limited application of transciptomic techniques is largely due to the inherent instability of mRNA, particularly in the presence of the extensive exogenous enzymes present within coral-derived samples.

Metabolomic techniques, which use NMR and mass spectroscopy to detect chemical fingerprints left behind by specific chemical processes, also show great promise for improving disease diagnosis and pathogen detection [86], [87]. While genomic, metagenomic, transcriptomic, and metabolomic approaches have the potential to generate extensive data, these techniques require expensive, specialized equipment and often the desired information is hidden within immense datasets that require specialized software and highly trained individuals to decipher. However, just as qPCR, which was only available to a handful of well-funded laboratories just a decade ago, is becoming increasingly affordable and accessible, the prohibitive cost of emerging technologies will certainly fall, increasing their availability to coral researchers.

### Validation of Diagnostics for Coral Pathogen Detection

The validity of any diagnostic test is determined by its ability to distinguish host organisms that have the disease from those that do not. Validity is comprised of two key components: sensitivity and specificity. Sensitivity describes the test's ability to correctly identify those with the disease and is expressed as the proportion of affected animals that are correctly identified as disease positive by the test compared to the total number of diseased animals tested. Specificity is the ability of the test to correctly identify those that do not have the disease and is expressed as the proportion of animals that are correctly identified as disease negative to the total number of disease-free animals tested [28]. In order to calculate the specificity and sensitivity of a test, we must first know which animals are actually infected with the disease. Such knowledge is usually gained by comparing a test's results with the results of a so-called gold standard, which theoretically has both a sensitivity and specificity of 100% [28]. For example, the gold standard for *Chlamydia* diagnosis in humans is isolation of the causative agent, the bacteria *C. trachomatis*. It is important to realize that while gold standards are the best evidence available, they are not infallible and gold standards providing full certainty are rare, particularly in a young field like coral disease research. Generally, the challenge is to find a standard that is as close as possible to the theoretical gold standard, but until effective gold standards are established for coral pathogen detection, it may be useful to use several of the diagnostic techniques described previously to cross-validate test results.

Coral researchers are faced with a unique set of challenges when developing disease diagnostics for the detection of specific pathogenic microbes among the diverse and complex coral holobiont. One major challenge is reproducibly obtaining high purity microbial DNA (or RNA) from coral-derived samples. The complex nature of the coral holobiont, which contains genetic material from the coral host as well as its associated algae, bacteria, and viruses, in combination with the presence of high concentrations of PCR inhibitors (e.g., salts and DNAses) make successful DNA extraction and pathogen detection from coral tissue extremely difficult. Several extraction methods have been developed to overcome these limitations, but consistently obtaining high quality DNA from coral samples remains a persistent challenge to coral researchers. Furthermore, there is the potential for gene copy number variability even between closely related bacterial strains as well as horizontal gene transfer between distantly related species, which could confound accurate detection and quantification. Early pathogen detection assays will therefore require extensive testing to confirm their specificity and sensitivity.

## Conclusions

Further development and application of diagnostic tools for coral pathogen detection is limited by a lack of knowledge of the organisms and genes involved in the onset and progression of most coral diseases. In particular, current knowledge of the causes of a large number of coral diseases is rudimentary, with only a few actual pathogens identified (reviewed in [7], [13]). Therefore, further research into coral disease ecology, in combination with robust biomedical approaches to describe diseases at gross and cellular levels is needed to develop an understanding of the pathogenesis of coral diseases and the interactions between agent, host, and the environment [18]. Only after pathogens are identified and their mechanisms of virulence determined can the development of diagnostics that target certain microbial groups or important genes proceed. Coral disease investigations, like other human, veterinary, or wildlife disease investigations, require an interdisciplinary approach, including the use of both traditional and developing technologies.

As coral diseases continue to threaten reefs worldwide, there is increasing urgency for tools to understand and control their spread. Several approaches, including phage therapy and probiotic addition, have been suggested to mitigate coral disease outbreaks [41]; however, the success of any of these strategies will depend upon rapid and reliable disease detection and diagnosis. With the extensive cost and potential environmental risk of certain control measures (e.g., phage therapy), it will be critical that diagnoses are made with an extremely high degree of certainty. Therefore, the development and testing of highly sensitive and specific coral disease diagnostics should be a major research priority. Accurate coral disease diagnosis will help to direct research and management strategies to address the true cause of disease on reefs and aid reef managers in their efforts to control the occurrence, prevalence, and severity of coral disease on reefs worldwide.
